# A review of fetal cardiac monitoring, with a focus on low- and middle-income countries

**DOI:** 10.1088/1361-6579/abc4c7

**Published:** 2020-12-18

**Authors:** Camilo E Valderrama, Nasim Ketabi, Faezeh Marzbanrad, Peter Rohloff, Gari D Clifford

**Affiliations:** 1 Data Intelligence for Health Lab, Cumming School of Medicine, University of Calgary, Calgary, AB, Canada; 2 Department of Biomedical Informatics, Emory University, Atlanta, GA, United States of America; 3 Department of Electrical and Computer Systems Engineering, Monash University, Clayton, VIC, Australia; 4 Wuqu’ Kawoq, Maya Health Alliance, Santiago Sacatepéquez, Guatemala; 5 Division of Global Health Equity, Brigham and Women’s Hospital, Boston, MA, United States of America; 6 Department of Biomedical Engineering, Georgia Institute of Technology, Atlanta, GA, United States of America

**Keywords:** fetal monitoring, low-and middle-income country, fetal cardiac assessment, mobile applications, perinatal care

## Abstract

There is limited evidence regarding the utility of fetal monitoring during pregnancy, particularly during labor and delivery. Developed countries rely on consensus ‘best practices’ of obstetrics and gynecology professional societies to guide their protocols and policies. Protocols are often driven by the desire to be as safe as possible and avoid litigation, regardless of the cost of downstream treatment. In high-resource settings, there may be a justification for this approach. In low-resource settings, in particular, interventions can be costly and lead to adverse outcomes in subsequent pregnancies. Therefore, it is essential to consider the evidence and cost of different fetal monitoring approaches, particularly in the context of treatment and care in low-to-middle income countries.

This article reviews the standard methods used for fetal monitoring, with particular emphasis on fetal cardiac assessment, which is a reliable indicator of fetal well-being. An overview of fetal monitoring practices in low-to-middle income counties, including perinatal care access challenges, is also presented. Finally, an overview of how mobile technology may help reduce barriers to perinatal care access in low-resource settings is provided.

## 1. Introduction

Perinatal complications account for 40% of the perinatal and maternal deaths worldwide (World Health Organization 2016a). Low-and middle-income countries (LMICs) contribute approximately 90% of total births, and 98% of the total perinatal deaths (World Health Organization 1996, Save the Children 2001, Blencowe *et al* 2016, Wang *et al* 2016).

The perinatal mortality rate is defined as the sum of the number of stillbirths and deaths occurring during the first seven days of life, per 1000 live births. In 2018, this rate stood at 19 per 1000 in LMICs, whereas in upper-middle and high-income countries, there was an average of seven and three deaths per 1000 live births, respectively (UNICEF *et al* 2018). The highest perinatal mortality rates have been reported for countries in Sub-Saharan Africa and South-Asia (28% and 26%, respectively) (UNICEF *et al* 2018) and may be underreported (Lopez *et al* 2007, Pattinson *et al* 2009). At the beginning of the twentieth century, the perinatal mortality rate in high-income countries (HIC) was as alarmingly high as it currently is in LMICs, but was effectively reduced by the expansion of antenatal care coverage, extended indications for Cesarean sections, and the introduction of perinatal screening technologies (cardiotocography (CTG), ultrasound, amnioscopy, amniocentesis, and pH-meter) (Dražančić 2001, Lawn *et al* 2009, Flenady *et al* 2011, Goldenberg *et al* 2016).

The most common causes of perinatal deaths are preterm birth-related complications (35%), intrapartum-related events (24%), and sepsis (15%) (UNICEF *et al* 2019). Studies conducted in LMICs have reported significant issues with prematurity, birth asphyxia, maternal hypertensive disorders, and septicemia being the most common causes of perinatal death (Allanson *et al* 2015, Mahdizadeh *et al* 2019). Fetuses and newborns are also disproportionately affected by infections, including syphilis, malaria, and animal and vector-borne diseases, leading to elevated mortality and morbidity (Han *et al* 2010, Goldenberg *et al* 2010).

Asphyxia, one of the most common causes of death during childbirth (Goldenberg *et al* 2007, Lawn *et al* 2009, Wall *et al* 2010, Vogel *et al* 2013), involves oxygen deprivation arising from obstruction of the placental blood flow, which may be rooted in maternal pre-eclampsia, placental abruption, or umbilical cord accident. The high death rate associated with asphyxia is mainly due to poor delivery management. Signs of asphyxia can be identified via fetal heart rate monitoring (Figueras and Gardosi 2011), and timely detection and intervention can reduce the risk of irreversible organ damage and identify cases requiring rapid deliveries (Goldenberg *et al* 2010). However, this basic monitoring procedure is not often practiced in LMICs.

Low birth weight (LBW), common among preterm (*<*37 weeks) or small-for-gestational-age (SGA) babies, is documented in 70–80% of the perinatal deaths (Lawn *et al* 2014, Allanson *et al* 2015, Mahdizadeh *et al* 2019). In LMICs, approximately 60% of LBW newborns are SGA (Lee *et al* 2013), which, in these countries, is often ascribed to intrauterine growth restriction (IUGR) (de Onis *et al* 1998, Lee *et al* 2013). IUGR can develop as a consequence of maternal vascular problems, malnutrition, or placental malfunction (Yakoob *et al* 2009).

While fetal cardiac assessment has been in use over the past four decades to diagnose, monitor, or predict adverse fetal conditions throughout pregnancy (Liston *et al* 2007, World Health Organization 2016b), there is still insufficient evidence with regards to its contribution to improved perinatal outcomes (Signore *et al* 2009). As a result, the World Health Organization (WHO) does not currently recommend continuous cardiotocography during labor for assessment of fetal well-being in healthy pregnant women undergoing spontaneous labor, but rather a periodic point of care auscultation (World Health Organization 2018a).

To explore the potential future directions for fetal monitoring in low-resource settings, this review presents an insight into fetal cardiac assessment, briefly explaining the affordability and applicability to each stage of pregnancy. Finally, we provide an overview of how mobile technology may reduce barriers to access perinatal care in poor-resource settings.

## 2. Fetal cardiac circulation

The human heart develops within the first six weeks of gestation (Gittenberger-de Groot *et al* 2019), with the majority of its functionality achieved by the eighth week of gestation (Archer and Manning 2009). The development begins with a primary heart tube, which evolves into the four-chambered adult heart structure, composed of two atria and two ventricles. Fetal circulation is unique in that blood is oxygenated in the placenta rather than in the lungs (Stock and Vacanti 2001).

The oxygenated blood from the placenta (umbilical vein) flows into the fetal liver and later, the inferior vena cava via the ductus venosus. On the one hand, the majority of oxygenated blood flows directly from the right atrium to the left atrium through the foramen ovale, and subsequently to the left ventricular to be pumped to the aorta (Stock and Vacanti 2001, Freeman *et al* 2012). On the other hand, the remaining oxygenated blood passes from the right atrium to the right ventricle and subsequently to the pulmonary vein. As fetal lungs are non-functional, a significant percentage of the blood in the pulmonary vein passes into the aorta via the ductus arteriosus (Stock and Vacanti 2001). The blood sent to the aorta circulates to the fetal brain and tissues. Finally, deoxygenated blood is transported to the placenta via two umbilical arteries (Freeman *et al* 2012). After birth, the foramen ovale closes, resulting in occlusion of the ductus venosus and arteriosus, and to the separation of the pulmonary and circulatory functionalities (Stock and Vacanti 2001).

### 2.1. Control of fetal heart rate

The fetal heart rate (FHR) represents the reciprocal of the interval between two successive fetal heartbeats. The heartbeats are controlled by cardiac muscle cells located in the myocardium (Stock and Vacanti 2001). The cardiac cells are categorized as myocardial contractile cells and myocardial conductive cells. The contractile cells stimulate the contractions required to pump blood throughout the body, whereas the conductive cells are the autorhythmic cells responsible for the heart’s electrical activity.

The majority of the conducting cells are located in the sinoatrial node (SAN), also called the pacemaker. The SAN initiates action potentials, resulting in the contraction of the atria at the onset of systole (Stock and Vacanti 2001). The action potential is propagated via the atrioventricular (AV) node to the bundle branches and Purkinje fibers located within the ventricular walls. This impulse initiates ventricular contractility, which, in turn, pumps blood to the pulmonary veins and the aorta, to mark the end of systole. The impulse then leaves the ventricles, marking the onset of diastole, during which the ventricular walls are repolarized. The electrical and mechanical events of a heart contraction generate the cardiac cycle, which is measured as the number of beats per minute (bpm).

Throughout pregnancy, the pace of fetal cardiac activity is controlled by the autonomic nervous system (ANS), baroreceptors, and chemoreceptors (Freeman *et al* 2012). The ANS is comprised of the sympathetic and parasympathetic nervous systems. The sympathetic system accelerates the heart’s electrical activity, yielding a faster FHR. The parasympathetic system, on the other hand, has the opposite effect on the FHR.

The balance between the sympathetic and parasympathetic nervous systems sets the baseline of the heart rate. However, as the sympathetic system matures earlier than the parasympathetic system, the FHR is higher in the first months of gestation. At 15 weeks gestation, the average FHR is 60 bpm. With the advancement of pregnancy, and the evolvement of the parasympathetic system, the FHR increases to approximately 110–160 bpm (Pildner von Steinburg *et al* 2013).

**Figure 1.**
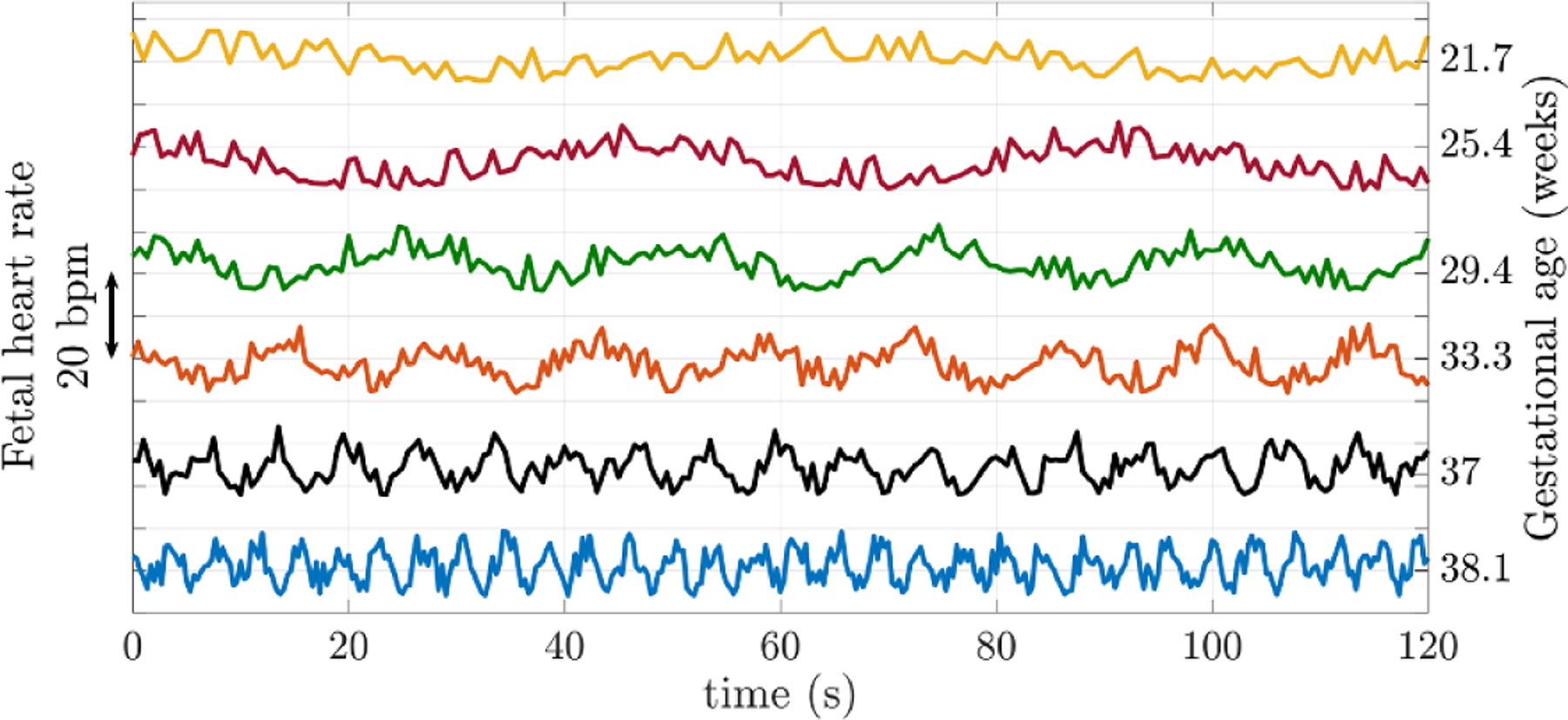
Illustration of the variation in FHR during gestation weeks 22–38. Note that the vertical axis has an arbitrary offset. FHR drops by about 15 bpm from week 25 to week 40 of gestation (Kapaya *et al* 2018). Adapted from Wakai (2004). Copyright © 2004 Elsevier Inc. All rights reserved.

Figure 1 illustrates how the sympathetic and parasympathetic systems affect the FHR across gestation, as reported by Wakai (2004). On analysis of traces of 61 healthy pregnant women, a short-term increase in FHR variability during the last trimester was noted. In contrast, long-term variability in FHR was most pronounced during the early gestational period.

The other two mechanisms that regulate the fetal heart rate are the baroreceptors and chemoreceptors. Barocepters are located in the aortic arch, carotid arteries, and brain stem. When blood pressure increases, baroreceptors signal the vagal nerve to slow down the heart rate, which then reduces blood pressure. In response to the blood pressure decrease, baroreceptors reduce the parasympathetic tone and stimulate an increase in the fetal heart rate and blood pressure.

The chemoreceptors, found in the aorta, carotid artery, and brain stem, impact the fetal heart rate via its oxygen level-sensing capacities. When the oxygen level decreases, the FHR is accelerated to increase the oxygen input rate from the placenta. However, when the oxygen level reduction is abrupt (hypoxemia), the chemoreceptors trigger a vagal response, resulting in a reduction in heart rate and an increment in blood pressure.

## 3. Fetal heart monitoring techniques

Fetal heart monitoring technologies can be categorized as either intermittent auscultation (IA) or electronic fetal monitoring (EFM) methods. IA techniques focus on verifying fetal cardiac performance by counting the number of beats over short periods, most commonly measured with a Pinard fetoscope, DeLee fetoscope, or hand-held Doppler device Blix *et al* (2019). EFM methods identify fetal stress or distress based on FHR variability, commonly performed via cardiotocography (CTG) (Miller *et al* 1984). It provides continuous information on the FHR, for a period of 10–60 min, using autocorrelation to obtain the average FHR over a specific window, which is generally every 3.75 s (Martis *et al* 2017).

EFM techniques can be categorized into invasive and non-invasive methods. In the invasive mode, the fetal electrocardiogram (fECG) is taken directly from the fetal scalp (Bakker *et al* 2004). Although the invasive technique is more accurate than non-invasive modalities, its use is limited to the intrapartum period, when the membranes are ruptured. In contrast, non-invasive methods are only employed during the antenatal period. Non-invasive methods extensively described in the literature include CTG, abdominal fECG, phonocardiography (fPCG), and fetal magnetocardiography (fMCG) (Gan *et al* 2009, Sameni and Clifford 2010), Wolfberg 2012, Ayres-de Campos *et al* 2015, Adithya *et al* 2017).

### 3.1. Fetal phonocardiogram

The fetal phonocardiogram (fPCG) is an electronic extension of the Pinard and DeLee stethoscopes. Similar to the stethoscope, fPCG is an IA technique in which a microphone is placed on the maternal abdomen to listen to fetal heart sounds (Sameni and Clifford 2010). The audible heart sounds correspond to the closure of the fetal valves during the cardiac cycle (Kovács *et al* 2011). The closure of the mitral and tricuspid valves generates a sound called S1, and the closure of the semilunar valves (pulmonary and aorta) generates a sound called S2. Both S1 and S2 have low acoustic energy and are affected by noises such as environmental noise, as well as other maternal and fetal physiological sounds, such as breathing, fetal movements, and maternal circulation (Várady *et al* 2003). The technique can extract cardiac timing and intensity of fetal heart sounds, which can carry useful diagnostic information (Adithya *et al* 2017).

The fPCG can be used during the antepartum phase (gestational week *≥*24) (Várady *et al* 2003). Although fPCG is an alternative to the traditional ultrasound used in perinatal management (Hamelmann *et al* 2019), it is underutilized (Adithya *et al* 2017) and suffers from significant challenges related to signal acquisition and processing. Further research is needed to improve fPCG to compete with standard fetal monitoring methods, i.e. CTG and ultrasound imaging.

### 3.2. One-dimensional doppler ultrasound

One-dimensional Doppler ultrasound (1D-DUS) estimates FHR by measuring the Doppler shift between ultrasound beams transmitted and received from the mechanical heart movements and blood flow. The Doppler magnitude frequency shift fD, is described as (Kohler and Sumner 2014):

(1) $$f_{D}=\frac{2f_{o}}{c}V\cos\theta ,$$

where *f*_*D*_ is the measured change in frequency (Hz), *f*_*o*_ the frequency of emitted ultrasound transducer in Hz, *c* the speed of sound in soft tissue in m s^−1^, *V* the velocity of the reflecting interface in m s^−1^ and *θ* is the angle between the ultrasound beam and the surface in radians.

The transmitted beam travels across various anatomical structures, from the skin surface, through the maternal skin and subcutaneous tissue, and then finally reaches the uterine muscles, the amniotic sac, and the fetal heart (Marieb and Hoehn 2007). The fetal heart movement reflects the ultrasound beam, and propagates the ultrasound waves in the reverse order. The distance between the DUS transducer and fetal heart depends on the maternal phenotype, which varies among nationalities (World Health Organization 2014a), socioeconomic status, as well as body mass index (Morgenstern *et al* 2009).

The shifted Doppler frequency is usually demodulated via the phase-quadrature demodulation, in which the received signals are mixed with the carrier signals sin 2*πf*_*o*_*t* and cos 2*πf*_*o*_*t* (Evans 1989). The demodulated signal is then autocorrelated to estimate the cycle period of the heartbeat rhythm (Shakespeare *et al* 2001).

Doppler ultrasound includes two different modes—the continuous wave (CW) and the pulsed-wave (PW). In CW, two piezoelectric crystals continuously monitor the reflection of the emitted wave. In PW, one piezoelectric crystal alternates between sending and receiving the sound waves. The dual functionality of CW Doppler enables the measurement of higher velocities. However, as velocities are measured in the same line of interrogation, it is impossible to know the origin of the velocity. In contrast, PW measures slower velocities, but the emitted sound waves are associated with the received waves, thus enabling the detection of the structure’s distance reflecting the wave.

CW is primarily integrated in hand-held Doppler transducers, while PW is used in standard CTG machines. Hand-held Doppler transducers are used during the intrapartum and antepartum periods after the 20th gestational week, to measure heart rate variability metrics as an indication of fetal wellbeing.

Mahomed *et al* (1994) carried out a randomized, controlled trial that showed that fetal monitoring using hand-held DUS transducers could detect a similar number of prolonged or late decelerations as ultrasonography. Additionally, the hand-held Doppler devices detected a substantially larger number of late and prolonged decelerations than the Pinard stethoscope. The potential of hand-held Doppler devices has also been reported by Devane *et al* (2017), following a randomized controlled study that demonstrated that they provide the same level of safety for screening and monitoring as cardiotocography in low-risk pregnancies.

### 3.3. Cardiotocography

Cardiotocography (CTG) is the simultaneous and continuous measurement of FHR and uterine pressure, often detected as uterine contractions (UC), and is a standard method for assessment of fetal wellbeing (Grivell *et al* 2015). To record the FHR, the medical assistant applies a gel on the maternal abdomen and the ultrasound transducer. The transducer is moved across the maternal abdomen while the technician listens for an audible version of the Doppler signal, in an attempt to identify the spot with maximum fetal heart rate impulse (as opposed to maternal arterial flow) (Freeman *et al* 2012).

CTG is used for fetal monitoring starting at the 20th week of gestation (Devane *et al* 2017), but most commonly indicated after the 28th week of gestation (Schneider and Maternal Fetal Medicine Study Group 2014). Although CTG is widely used, it suffers from high intra- and inter-interpreter variability (Todros *et al* 1996, Bernardes *et al* 1997, Blix *et al* 2003, Blackwell *et al* 2011, Hruban *et al* 2015), resulting in low specificity. To reduce this subjectivity, Dawes *et al* (1992) introduced a computerized version of the CTG. A Cochrane review of two studies (469 subjects) concluded that the mortality rate in a population monitored by computerized CTG was four times lower than in the population monitored by visual CTG (0.9% vs. 4.2%) (Grivell *et al* 2015).

Computerized CTG has also been used in recent years to develop artificial intelligence methods to detect abnormal FHR patterns, achieving comparable results to clinical assessment of the CTG (Chudáček *et al* 2011, Warrick *et al* 2012, Georgieva *et al* 2019). Notably, these artificial intelligence-based CTG systems have shown the potential to discriminate between normal and IUGR fetuses (Stroux *et al* 2017, Signorini *et al* 2020).

Although CTG is a standard method used for fetal monitoring in high-income countries, controlled clinical trials have not provided evidence of its benefits; CTG was associated with a 20% increase in Cesarean interventions with no improvement in fetal outcomes (Devane *et al* 2017). In addition, the use of CTG was not associated with statistically significant improvements in perinatal outcomes as compared to traditional intermittent auscultation methods (Kamala *et al* 2018b, Mdoe *et al* 2018b).

#### 3.3.1. The nonstress test

The nonstress test (NST) monitors FHR patterns for at least 20 minutes, and is designed to identify accelerations associated with fetal movements (Malhotra *et al* 2014). This test calculates the baseline FHR, which is later used to measure long-term and short-term variability, episodes of high and low variation, acceleration, and deceleration. Test results are considered normal (reactive) when more than two accelerations occur within 20 minutes of observation. In contrast, a non-reactive result is when, no more than one acceleration occurs within 40 minutes (American College of Obstetricians 2000). The NST has a low false-negative (0.3%), but a high false-positive (50%) rate (Eden *et al* 1990). Of note, the test carries no risk of inducing any uterine contractions.

#### 3.3.2. Contraction stress test

The contraction stress test (CST) is based on the premise that contractions, induced using oxytocin or nipple stimulation (Malhotra *et al* 2014), trigger a hypoxic state (Signore *et al* 2009). A healthy fetus can tolerate this hypoxic state, whereas a non-healthy fetus will respond with late FHR decelerations (Malhotra *et al* 2014). Although this method has a low false-negative rate (0.04%) (Freeman *et al* 1982) and a lower false-positive rate than NST (30% vs. 50%) (American College of Obstetricians 2000), it requires an intravenous intervention, which increases the risk of fetal hypoxia and of induction of preterm birth (Malhotra *et al* 2014).

#### 3.3.3. Acoustic stimulation

Acoustic stimulation is a variation of NST, in which if there are no fetal movements, vibroacoustic stimulation (usually with a laryngeal stimulator) may be activated for 3 seconds on the maternal abdomen over the fetal head. This is performed to ‘awaken a sleeping fetus’, before initiation of the NST (Malhotra *et al* 2014). The artificial larynx produces a vibratory stimulus of 80 Hz that causes a healthy fetus to increase physical activity, as measured by an increase in FHR. Its advantages include shortening of the NST by 10 minutes (Clark *et al* 1987), and reduction of the number of non-reactive states, without affecting readability (Smith *et al* 1985, Smith *et al* 1986). In cases of a non-reactive result, the acoustic stimulation is repeated for 5 min. If the test is still non-reactive, a fetal biophysical profile or CST is indicated (Malhotra *et al* 2014).

### 3.4. Fetal electrocardiogram

The fECG records the complex electrical activity of the fetal heart. The main components of the ECG signals are P, Q, R, S, and T waves. The P wave represents atrial depolarization, which is followed by the atrial contraction (atrial systole). The atrial contraction is extended to the QRS complex, which corresponds with ventricular depolarization, with ventricles contracting at the peak (R wave). The ventricular contraction lasts until the ST-T wave, which corresponds with ventricular repolarization and relaxation. fECG can be captured in an invasive manner during the intrapartum period, when the cervix is dilated, and the fetus scalp is visible, or in a non-invasive manner, starting from the second trimester. The fECG is also used to complement CTG at intrapartum to reduce unnecessary Cesarean sections (Vijgen *et al* 2011, East *et al* 2014).

#### 3.4.1. Invasive fetal electrocardiogram

The invasive fetal electrocardiogram (invasive-fECG) requires the rupture of the membranes to introduce electrodes, via the cervix, and to place them on the fetal scalp (Hon 1960, Hon and Hess 1960, Hon and Lee 1964). This technique processes the recorded signals to visualize the P and T waves, as well as the QRS complexes.

Scalp fECG has been used as a complementary technique during intrapartum FHR monitoring (Amer-Wåhlin *et al* 2001, Norén *et al* 2006). The morphology of the ST segment is analyzed to find patterns associated with uterine complications (Larks and Longo 1962, Larks and Larks 1966). Invasive fetal ST can be captured and analyzed from the 36th gestational week and is indicated in high-risk pregnancies when a non-reactive CTG is obtained, or labor is induced by oxytocin. Although its use has shown to effectively reduce neonatal encephalopathy (Norén *et al* 2003, Norén *et al* 2006), randomized control trials of this technology have yet to demonstrate a clear benefit.

#### 3.4.2. Non-invasive fetal electrocardiogram

Non-invasive fECG measures the electrical activity of the fetal heart via electrodes which are placed on the maternal abdomen (Lai and Shynk 2002). This technique is indicated from the 18th week of gestation (Sameni and Clifford 2010), and therefore has much wider applicability than invasive ST analysis and can replace Doppler auscultation for the fetal heartbeat.

Although abdominal fECG signals have a relatively low amplitude (microvolts), it can provide a more accurate estimate of beat location when compared to the CTG, and hence a more accurate quantification of fetal heart rate variability indexes (Jezewski *et al* 2012, Clifford *et al* 2014, Jezewski *et al* 2017). The morphology and beat-to-beat heart rate variability estimated from fECG are established indicators of pre-eclampsia and IUGR. For instance, a study conducted on 106 patients (30 healthy, 44 mild pre-eclampsia, and 32 severe pre-eclampsia subjects) at 34–40 weeks of gestation, reported that FHR variability indices were associated with the suppression of fetal biophysical activity and the development of fetal distress in women suffering from severe pre-eclampsia Lakhno (2017). Similarly, Velayo *et al* (2017) assessed the impact of IUGR on FHR variability indices extracted from abdominal fECG recordings of 20 control and 15 IUGR singleton pregnant women. While the authors identified clear P-QRS-T complexes in all cases, prolonged QT intervals were measured in IUGR fetuses.

Over the last 30 years, a variety of methods have been proposed for extracting and processing fECG signals (Sameni and Clifford 2010), Jaros *et al* 2018). Methods range from adaptive filtering (Park *et al* 1992, Shao *et al* 2004, Martens *et al* 2007) to non-adaptive approaches such as, independent component analysis (Sameni *et al* 2006), principal component analysis (Al-Zaben and Al-Smadi 2006), wavelet transforms (Castillo *et al* 2013, Wu *et al* 2013) and neural networks (Assaleh 2007, Amin *et al* 2011, Behar *et al* 2014). Many of these techniques suffer from significant limitations due to causality and signal stability. Other approaches based on generalized eigenvalue decomposition have shown more promise (Sameni *et al* 2007). In Clifford *et al* (2009) and Behar *et al* (2016), it was shown that this approach was able to accurately resolve both QT interval and ST elevation/depression from non-invasive fECG. However, this promising result has yet to be applied in a randomized clinical trial to demonstrate efficacy.

### 3.5. Fetal magnetocardiography

Fetal magnetocardiography (fMCG) uses a sensitive, superconducting sensor to measure the magnetic field of fetal heart activity (Kariniemi and Hukkinen 1977, Sameni and Clifford 2010), Kiefer-Schmidt *et al* 2012). The fMCG provides a waveform almost identical to that of fECG, but at a higher signal-to-noise ratio, and with a higher resultant quality of waveform (Peters *et al* 2001, Kiefer-Schmidt *et al* 2012). The higher quality enables the classification of arrhythmias and detection of congenital heart diseases (Kähler *et al* 2001), as well as the ability to assess fetal neurological development (Wakai 2004).

The fMCG is used from the 20th gestational week (Peters *et al* 2001). Yet, although it provides good-quality waveforms, it is not routinely used in perinatal care due to its higher costs, i.e. the need for a shielded room, and highly skilled personnel (Peters *et al* 2001, Kiefer-Schmidt *et al* 2012). Alternative methods, such as the abdominal fECG or the hand-held Doppler, can be used at any time during pregnancy, and can even be performed at home by the patients themselves (Sameni and Clifford 2010).

## 4. Ultrasound imaging

Ultrasound imaging is considered the gold standard for fetal monitoring in high-income countries (Liston *et al* 2007, World Health Organization 2016c). It evaluates fetal growth, fetal cardiac structure and function, and fetal, uterine, and placental blood circulation. Ultrasound imaging is usually indicated in the second trimester, particularly after the 20th week of gestation, with a scan recommended by the WHO before week 24 (Whitworth *et al* 2015, World Health Organization 2016c, World Health Organization 2016b).

Ultrasound imaging is known to effectively assess pregnancy viability, estimate gestational age, detect multiple pregnancies, and determine placental position (Whitworth *et al* 2015). While there is no compelling evidence that ultrasound scans reduce perinatal mortality (Neilson 1998, Dražančić 2001), they can be used to validate suspicious diagnoses without invasive and risky interventions, reduce labor induction for post-term pregnancy, and detect fetal malformation (Whitworth *et al* 2015). Moreover, a review of 58 obstetric articles, concluded that ultrasound imaging provides appropriate clinical management in at least 30% of cases when used by skilled operators (Groen *et al* 2011).

Ultrasound imaging has also shown potential in the assessment of IUGR. A comparison between 38 IUGR and 32 appropriate for gestational age (AGA) fetuses showed that growth-restricted fetuses had a statistically significant thicker aortic wall than the AGA fetuses (1.9 mm vs. 1.15 mm) (Cosmi *et al* 2009). The median diameter of the abdominal aorta was also significantly higher in IUGR than in AGA fetuses. The thicker aortic wall in the IUGR fetus was also noted by Gomez-Roig *et al* (2015), who compared 35 IUGR fetuses with 49 AGA fetuses. In contrast to Cosmi *et al* (2009), they reported on the substantially lower diameter of the abdominal aorta for fetuses with IUGR (Gomez-Roig *et al* 2015).

There is no scientific evidence for, or consensus on how often ultrasound scans should be performed during pregnancy. Some obstetricians recommend at least four ultrasound scans during normal pregnancies, whereas others recommend only one, to be performed before the 24th gestational week (Papp and Fekete 2003). When four scans are performed during pregnancy, the first is conducted between weeks 10 and 14, to validate the pregnancy and estimate gestational age. The second scan is carried out between weeks 18 and 22, to detect fetal anomalies and confirm gestation age. The third scan is scheduled between weeks 30 and 34 of gestation, to assess fetal growth. The final scan is scheduled between weeks 36 and 38, and focuses on the fetal weight, position, and orientation/presentation, which helps to determine the optimal mode of delivery.

### 4.1. Fetal biometry

Ultrasound imaging enables measurement of different fetal organs, and estimation of gestational age and fetal weight. The most common measures are biparietal diameter (BDP), femur length (FL), head circumference (HC), crown rump length (CRL), and abdomen circumference (AC) (Malhotra *et al* 2014). Using a combination of these measurements, fetal weight estimates are within 5% of the actual weight in 50% of cases, and within 10% in 80% of the evaluations (Galan *et al* 2002).

Fetal biometry measurements have been shown to be more accurate during the first trimester. During the second and third trimesters, fetal measurement accuracy is impacted by genetic and nutritional factors (Reece *et al* 1989). Recently proposed formulas combining the transcerebellar diameter (TCD) with FL and AC are emerging as a solution for dating late pregnancies (after the 24th gestation week), with gestational age estimates within *±*3 weeks of the CRL measurement taken between the 8th and 14th gestational weeks (Deb *et al* 2020).

Fetal biometry measurements can differentiate between fetuses that are IUGR and those that are constitutionally small (SGA) (Soothill *et al* 1993). Specifically, when an estimated weight is below the 10th percentile for gestational age, the fetus is considered growth-restricted, as defined by the American College of Obstetricians and Gynecologists (ACOG) guidelines (Uquillas *et al* 2017). However, a previous study, conducted by the Prospective Observational Trial to Optimize Pediatric Health (PORTO), found that only 2% of fetuses whose estimated birth weight was within the 3rd and 10th percentile, had an adverse perinatal outcome; the authors concluded that the threshold should be below the 3rd percentile (Unterscheider *et al* 2013).

Furthermore, fetal biometry measurements ignore the fact that 10% of the normal population is genetically predisposed to be small, thus increasing the false-positive rate (Galan *et al* 2002). Hence, to increase the accuracy in detecting IUGR, fetal biometry should be combined with methods assessing the fetal ANS physiology (Galan *et al* 2002). When IUGR is detected, the pregnancy is categorized as high-risk, as the condition has long-term consequences.

### 4.2. Doppler velocimetry

Doppler velocimetry assesses the blood flow in the umbilical arteries and vein to evaluate pregnancies at risk of fetal compromise (Trudinger *et al* 1986), such as growth restriction (Alfirevic and Neilson 1995) or cardiovascular abnormalities (Berkley *et al* 2012). In healthy pregnancies, the placental and fetal circulation transfers oxygen and nutrients, and eliminates fetal waste products (Malhotra *et al* 2014).

Umbilical flow is assessed using different indexes, such as systolic and diastolic ratio, pulsatility index and resistance index (American College of Obstetricians 2000). Higher indexes indicate significant vascular resistance, thus implying that fetal health is at risk (Berkley *et al* 2012, Signore *et al* 2009).

The resistance indexes are mainly measured on the umbilical artery (UA), the middle cerebral artery (MCA), and the ductus venosus (DV) (Mone *et al* 2015). Of these three areas, the UA Doppler is the only device that has been the subject of randomized controlled trials, which have supported its feasibility for fetal surveillance in high-risk pregnancies (Alfirevic *et al* 2017). The UA Doppler measures the resistance in fetoplacental circulation flow, providing a pulsatility index (PI). In a healthy fetus, the UA has a forward flow. However, increases in placental resistance obliterate the muscular arteries in the placental villi, resulting in a reduced diastolic flow (Berkley *et al* 2012), which then eliminates and later reverses the fetoplacental circulation flow. Both the absence and the reversal of flows can be visualized in the Doppler images. In the case of the absent end-diastolic flow (AEDF), the pronounced systolic peak is followed by an interruption, while in the reversed end-diastolic flow (REDF), the systolic peak is followed by a negative peak. In fetal growth-restricted pregnancies with AEDF or REDF, delivery is recommended at week 32 (Royal College of Obstetricians and Gynaecologists 2002).

Randomized controlled trials have demonstrated that a UA PI greater than the 95th percentile in restricted-growth fetuses is an indicator of a perinatal adverse outcome (Unterscheider *et al* 2013, O’Dwyer *et al* 2014). The use of UA Doppler was also shown to be effective in reducing the incidence of perinatal deaths and induced deliveries (Alfirevic *et al* 2017). MCA flow can be used to detect problems caused by fetal hypoxemia in IUGR. In a hypoxic state, most of the oxygenated blood is supplied to the brain, heart, and adrenal glands, affecting the peripheral circulation (Uquillas *et al* 2017). This phenomenon is called *brain-sparing reflex* and is observable in the waveform of the MCA Doppler. MCA Doppler is also a reliable indicator of anemia. Moreover, the MCA PI/UA ratio can indicate adverse perinatal outcomes (Mari and Hanif, 2008), which are related to an increment of the diastolic flow due to hypoxia (Morris *et al* 2012).

DV flow can be used to detect the cardiac failure in IUGR, particularly in cases of early-onset fetal growth restriction (Baschat 2010). It is a reliable marker of acidemia and stillbirth (Baschat 2010), which are caused by absent or reversed end-diastolic pressure at the ductus venosus. Although DV flow measurement displays moderate accuracy in detecting fetal compromise, previous works have suggested that DV Doppler alone is insufficient for fetal surveillance (Royal College of Obstetricians and Gynaecologists 2002). Furthermore, DV Doppler does not offer any added benefit over traditional CTG for fetal monitoring (Lees *et al* 2015). Nevertheless, delaying delivery until finding an abnormality using DV flow could prevent neurological impairment in the long-term (Ganzevoort *et al* 2017). Randomized controlled trials are still needed to more accurately assess the benefits of DV flow measurement.

Other anatomical areas useful in the management of fetal growth-restricted pregnancies are the uterine artery, the aortic isthmus, umbilical vein, and the atrioventricular valves (Mone *et al* 2015). The uterine artery flow is useful in identifying pre-eclampsia and SGA neonates in high-risk pregnancies (Royal College of Obstetricians and Gynaecologists 2002). The aortic isthmus measures the balance between the brain’s impedance and systemic circulation, indicating cardiac dysfunction when there is an abnormal balance (Cruz-Martinez *et al* 2011). Umbilical vein flow provides an indication of fetal venous circulation, where high values suggest increased venous pressure that results in right-sided heart failure and myocardial hypoxia (Nicolaides *et al* 2002).

### 4.3. Fetal echocardiography

Fetal echocardiography is a non-invasive ultrasonography technique that examines fetal cardiac anatomy and function (Godfrey *et al* 2012). The accuracy and speed of performing fetal cardiac assessment have improved in the last decades, following the introduction of advanced techniques such as color Doppler (DeVore *et al* 1987). The primary use of fetal echocardiography is in the detection of congenital heart diseases (CHDs), which are the most common abnormality in fetuses, with a prevalence of around 8 to 9 per 1,000 live births (Hoffman and Christianson 1978). The procedure assesses the heart structure, as well as the direction, pattern, volume, and velocity of flow (Allan 1986). The basic visualization of the chambers can be extended to include blood flow through the chambers, using a technique called ‘five chambers views’, which increases the sensitivity of detecting CHDs by 5%, achieving a final sensitivity rate of 65% (Allan 2000).

Fetal echocardiography also includes a pulse wave Doppler component, which is recommended for a complete evaluation of the fetal heart. The pulse wave shows the blood flow through the atrioventricular, mitral, and tricuspid valves (Abuhamad and Chaoui 2012). These valves generate a dual-peak Doppler waveform that comprises the E-wave, which is the passive diastolic filling, and the A-wave, which is the active diastolic filling (‘atrial kick’) (Abuhamad and Chaoui 2012). In healthy fetuses, the amplitude of the A-wave is greater than that of the E-wave, which increases throughout gestation. A higher increase in the E-wave/A-wave ratio is a sign of IUGR or congenital cystic adenomatoid malformation, which can lead to mitral or tricuspid regurgitation (Mari and Hanif 2008, Mäkikallio *et al* 2008).

Modern echocardiography techniques include three-dimensional (3D) and four-dimensional (4D) fetal heart assessment (Deng *et al* 1996), which enable real-time examination of the heart rate function, and a more accurate assessment of the heart structures (Chaoui *et al* 2004, Bennasar *et al* 2010, Ionescu 2010).

Although fetal echocardiography is considered one of the most relevant fetal cardiac assessment techniques, it is costly and requires qualified specialists to perform the examination (Caserta *et al* 2008). Therefore, fetal echocardiography is only provided when indicated by specific maternal and fetal conditions.

## 5. Comparison of fetal cardiac monitoring methods

Table 1 presents an comparison of the fetal cardiac monitoring techniques presented in sections 3 and 4, particularly with respect to the following four criteria:

- medical equipment cost;
- operator training requirement;
- gestational week at which the device can be used; and
- evidence supporting the device’s utility.

### 5.1. Cost analysis and availability in LMICs

The fMCG is an expensive method, requiring specialized operator training, dedicated shielded rooms, each costing approximately $350 000 to construct (Strasburger *et al* 2008) and high maintenance (Peters *et al* 2001, Cannie *et al* 2006, Kiefer-Schmidt *et al* 2012), which have limited its use in HICs (Cannie *et al* 2006, Wakai 2014) and its introduction into and widespread integration in LMICs.

Even compact portable ultrasound equipment, such as the GE LOGIQ Book XP (General Electrics, Milwaukee, WI, USA), costs at least $10 000, and carries additional expenses such as maintenance, supplies, battery replacement, and staff training (World Health Organization 2014b). However, a recent review on the use of ultrasound in LMICs reported an expanding utilization of low-cost, portable imaging technology in low-resource settings (Stewart *et al* 2020).

The ultrasound devices most commonly used for fetal monitoring were provided by Sonosite Inc (Bothell, WA, USA) (Kimberly *et al* 2010, Greenwold *et al* 2014, Boamah *et al* 2014, Kozuki *et al* 2016) and General Electric (Ome-Kaius *et al* 2017, Goldenberg *et al* 2018). Despite these early indications of their increasing integration in fetal monitoring in LMICs, there is still a need to assess the benefits, trade-offs, and potential drawbacks of large-scale obstetric ultrasound implementation in these regions (Kim *et al* 2018).

Several randomized controlled studies using ultrasound imaging in antenatal care in LMICs, did not show any significant reductions in adverse perinatal outcomes (Neilson 1998, Dražančić 2001, Kim *et al* 2018, Goldenberg *et al* 2018, Franklin *et al* 2018). Moreover, this technique requires specialized skilled operators, who are in limited supply in LMICs (World Health Organization 2014b).

Scalp fECG is limited to intrapartum use and requires specialized training. The most common device used for invasive fECG is the STAN monitor (Neoventa Medical, Goteborg, Sweden) (Clifford *et al* 2014). Vijgen *et al* (2011) reported that the average cost of ST analysis in 2 827 deliveries in a Dutch hospital was €1345, which is not feasible for LMICs.

Abdominal fECG can be captured using low-cost equipment, which does not require skilled users (Behar *et al* 2016, Marzbanrad *et al* 2018). The Monica AN24 monitor (Monica Healthcare, Nottingham, UK) and the Meridian M100/M1000 monitors (MindChild Medical, North Andover, MA), both which have been approved by the Food and Drug Administration (FDA) and the European Commission (CE), are two commercial devices commonly used for non-invasive fECG (Behar *et al* 2016). However, although the cost of fECG devices is relatively low ($4500 for the Monica Novii Wireless Patch System (Cardo Medical 2020)), non-invasive fECG is still not widely used since the systems still require further testing to definitively demonstrate that the morphological analysis is similar to that provided by the scalp electrocardiography method (Smith *et al* 2019, Kahankova *et al* 2019).

On average, CTG machines cost at least $450 (World Health Organization 2016c) and require maintenance, supplies, and training, thereby limiting its use in low-resource settings (World Health Organization 2016b). Although obstetric protocols in HICs recommend CTG, its use in LMICs has not improved fetal outcomes in comparison to auscultation methods (Housseine *et al* 2018). In contrast, auscultation methods, particularly the fetoscope, have been shown to be associated with reduced perinatal deaths in LMICs (Wall *et al* 2010).

Of the various auscultation methods available, the Pinard stethoscope is the most available tool in resource-constrained regions due to its affordable cost (Jauniaux and Prefumo 2016). Before its introduction, midwives used a stethoscope in the labor ward to listen to the fetal heart rate for ten minutes every half hour (Mahomed *et al* 1994). However, auscultation with the stethoscope can be uncomfortable for both patients and practitioners. Additionally, the stethoscope provides unsatisfactory results due to confounding factors such as environmental noise (Mahomed *et al* 1994).

Hand-held Doppler transducers are simple to use and can be purchased for as little as $17 (Stroux *et al* 2016). They require less time than the Pinard stethoscope to assess the fetal heart rate (Mahomed *et al* 1994). Plotkin *et al* (2019) compared the performance of hand-held Doppler and Pinard devices for fetal monitoring in the intrapartum period by reviewing 19 studies conducted in India and African LMICs. The comparison showed that Doppler devices accurately detected more fetal abnormalities than the Pinard stethoscope. However, there was no statistically significant improvement in perinatal outcomes when an anomaly was detected. The authors suggested that the lack of progress resulted from the poor clinical management and protocol referral of abnormality events. The review also found that both patients and medical providers preferred Doppler hand-held devices than Pinard stethoscopes, thereby justifying the integration of Doppler in fetal monitoring protocols for LMICs.

## 6. Usage of devices in LMICs

In LMICs, there is limited availability of the life-saving and complex medical devices routinely used for fetal cardiac monitoring in developed countries. The technologies involve the use of ultrasound technology, telemedicine, CTG and other fetal monitoring techniques that may not be easily implementable in low-resource settings. However, multiple research studies have demonstrated the acceptable effectiveness of suitable and appropriate technologies in these regions for fetal cardiac monitoring (Banta and Thacker 2002, Wyatt 2008, Rahman *et al* 2012, Devane *et al* 2017, Alves *et al* 2019).

Mdoe *et al* (2018a) demonstrated the superiority of fetal heart rate monitoring using continuous Doppler when compared to intermittent fetoscope auscultation. In their study, there was an 8.1% incidence of abnormal fetal heart rate detection when using continuous Doppler, versus 3.0% with the use of intermittent auscultation.

Additional studies have provided evidence for the preferential use of Doppler ultrasound for fetal cardiac activity monitoring. It has also been shown to accurately determine the number of reported fetal demise and to classify them as stillbirths versus neonatal deaths (Lopez *et al* 2007). Additionally, the emotional reassurance among mothers associated with hearing the fetal heartbeat amplified by the Doppler is a positive experience that could influence positive outcomes. Compared to the Pinard stethoscope, the Doppler was noted to be superior in detecting abnormal intrapartum fetal heart rate but was not associated with improved perinatal outcomes (World Health Organization 2018a).

Britton *et al* (2019) demonstrated the use of ‘tele-ultrasound’ for fetal cardiac monitoring in low resource settings. In the study, fetal monitoring devices were delivered to participants, to overcome challenges of geographical distance, lack of facilities and inadequate healthcare personnel that are common in these areas. The ‘tele-ultrasound’ technique was found to be low-cost, and reliable and implementable in resource-limited settings.

World Health Organization (2018b) reported on improved perinatal outcomes in following the use of CTG during labor in LMICs. Others have associated CTG (cardiotocography) with higher Cesarean section rates, with no added benefit on perinatal outcomes, and therefore do not recommend its use in low-resource settings (Housseine *et al* 2019). High-quality evidence considering implementation barriers and enablers is needed to determine the optimal fetal monitoring technique in Low-resource settings. World Health Organization (2018b) noted that there are significant gaps between international recommendations and what is practically possible in most resource-constrained countries.

A study in Uganda highlighted critical challenges as shortage of staff and devices, institutional challenges, and maternal perceptions to monitoring Munabi-Babigumira *et al* (2019). Another study in Tanzania listed lack of strict protocol for use and misidentification of maternal heart rate as the challenges associated with the introduction of Moyo, an electronic strap on a fetal heart rate monitor (Lafontan *et al* 2019) developed to improve intrapartum fetal heart rate monitoring. More specifically, the quick, short, and unstructured assessments and inferences limited the initiation of various interventions due to indecisiveness. The introduction of CTG was associated with simpler and more efficient monitoring of labor, but no improved outcomes (Kamala *et al* 2018a, Lafontan *et al* 2019).

## 7. Telemonitoring for perinatal care, an alternative for LMICs

In recent years, telemonitoring applications have been developed to enhance maternal and fetal monitoring. These applications have been made possible by the high penetration of mobile telecommunications technologies in LMICs, with approximately 90% of the population owning a mobile phone device (Bergström *et al* 2015). This high coverage of mobile phone access can be exploited to overcome perinatal care access barriers in LMICs, such as low literacy levels, poor road infrastructure, and medical professionals and equipment deficiencies (Eysenbach and Consort-EHEALTH Group 2011, Sondaal *et al* 2016, Hasan *et al* 2017).

The feasibility of mobile health applications (mHealth) in improving antenatal care was presented by Feroz *et al* (2017) after reviewing 14 cases conducted in sub-Saharan Africa, Southeast Asia, and Middle-East countries. The authors found that mHealth solutions can improve perinatal care services by increasing the percentage of women attending the minimum WHO-recommended perinatal visits. They noted that the most effective mobile apps were those that used client education and behavior change communication via short messages and patient tracking to allow for patient follow-up in subsequent visits.

In a review of telemonitoring in obstetrics, researchers reported that mobile applications connected to external devices, such as electrodes, body sensors, and thermometers provided effective maternal and fetal monitoring (Alves *et al* 2019). The used external devices enabled the digitalization of data, which was later analyzed by medical professionals or artificial intelligence techniques to detect abnormalities. Among the available fetal monitoring mobile applications, Jatmiko *et al* (2015) developed a method to identify the location of the fetus from an ultrasound image. The algorithms tested with pictures taken in a public hospital in Indonesia, demonstrated 93% accuracy in the detection of the fetal head and abdomen. Similarly, Khan *et al* (2016) developed methods to calculate the mean abdominal diameter (MAD) from an ultrasound image. A mobile app prototype was tested on ultrasound images captured by professional midwives in a Norwegian hospital, and demonstrated a mean error of −0.06 mm.

Awiti *et al* (2016) developed an Android-based digital fetoscope prototype. Fetal heart sounds are acquired using a Pinard horn and a microphone and are later sent to a smartphone via Bluetooth technology. In the smartphone, the audio signals are processed to display a heartbeat. When testing the system on adults, and comparing the measurements with those of a standard electronic sphygmomanometer, the Android-based digital fetoscope achieved a root mean square error of 7.23 bpm with a standard deviation of 5.44 bpm.

Tapia-Conyer *et al* (2015) introduced a mobile maternal-fetal monitoring application in a resource-poor and educationally-limited community in Mexico. The project aimed to evaluate the feasibility of providing remote antenatal care. The staff in the rural medical center was trained to use the mobile fetal monitor, which comprised a fetal ultrasound heart monitor, a uterine tocodynamometer, and additional tools for recording maternal blood pressure, blood glucose, and urinary protein values. The researchers split the 125 volunteers into control and study groups. The study group received perinatal care at the local medical center using the mHealth system, whereas the control group received standard perinatal care at the main public hospital. Tapia-Conyer *et al* (2015) observed that volunteers using the mHealth system were more than twice as likely to adhere to antenatal care monitoring than those receiving standard of care. There were no statistically significant differences in adverse perinatal outcomes between the two groups, suggesting that the tested mobile technology did not compromise maternal and fetal health.

A low-cost fetal monitoring system introduced in a rural Guatemalan community in 2013, was later developed into a system that was tested in a randomized controlled trial in 2015–2017 (Stroux *et al* 2016, Martinez *et al* 2017, Martinez *et al* 2018). The mHealth system consists of a low-cost one-dimensional Doppler transducer and a blood pressure device connected to an Android-based smartphone running an app designed for low-literacy traditional birth attendants (TBAs). The TBAs were trained to use the mHealth system during home visits. When a TBA visited a patient, the app guided the TBA to find the fetus and record a Doppler recording of the fetal cardiac activity for up to 20 minutes. The TBA also recorded blood pressure readings from both arms. The app then guided the TBA through basic questions presented through appropriate pictograms and audio prompts, to assist in the identification of concerning signs and symptoms during pregnancy. In the event a risk factor was identified, the app connected the TBA to appropriate (local or remote) medical care through a voice call, to provide decisional support and onward referral to appropriate healthcare, if needed. The Doppler signal and maternal blood pressure recorded with the mHealth system has allowed the development of different modules for providing estimates of fHRV, gestational age, and hypertension (Valderrama *et al* 2017, Valderrama *et al* 2018, Valderrama *et al* 2019, Valderrama *et al* 2020a, Valderrama *et al* 2020b, Katebi *et al* 2020).

## 8. Discussion and conclusion

Fetal monitoring is performed with a variety of devices and approaches, with CTG and ultrasound imaging considered the ‘standard of care’ in high-income countries. Despite the paucity of evidence supporting the utility of these techniques in reducing perinatal mortality and morbidity (Neilson 1998, Devane *et al* 2017), their use may still be beneficial throughout pregnancy. Specifically, CTG may facilitate the detection of signs of hypoxia requiring Cesarean delivery (Grivell *et al* 2015), and ultrasound imaging can help estimate gestation age before gestational week 24, and to detect multiple pregnancies and fetal abnormalities requiring vigilance or/and interventions (Groen *et al* 2011, Whitworth *et al* 2015). However, in resource-constrained settings, CTG and Doppler imaging are scarce, due to their high cost and the need for trained operators (Dražančić 2001).

Among fetal monitoring techniques, 1D Doppler transducers provide an affordable option, with an outstanding balance between cost, clinical utility, and operator learning curve, thereby making its use most practical (see table 1). Moreover, Doppler transducers have been shown to be comparable to CTG in fetal heart rate monitoring in LMICs. Taken together, Doppler transducers, which are widely available in these regions (Becker *et al* 2016), are a reliable ‘standard of care’ in low-resource regions (Devane *et al* 2017, Housseine *et al* 2018).

Perinatal care access in LMICs can be facilitated by telemetry (Bergström *et al* 2015). Mobile technology can support fetal monitoring analysis and transmission of clinical information collected using low-cost devices, such as Doppler transducers, portable CTG, or auscultation methods. In this manner, economic and geographical barriers can be overcome to increase perinatal care coverage in LMICs.

The feasibility of the use and impact of mHealth mobile applications in fetal monitoring has been shown in several works conducted in LMICs (Tapia-Conyer *et al* 2015, Stroux *et al* 2017, Feroz *et al* 2017, Martinez *et al* 2018). With the advent of increasingly complex smartphones, particularly those with embedded ‘AI’ chipsets), mHealth applications may extend beyond data collection and decision support systems, by processing complex maternal and fetal information in an edge computing paradigm, which allows the use of mobile applications without relying on network communication. However, regulation (especially in the US) is likely to limit this development, and to drive the solutions to higher-cost, self-contained devices.

Increasingly higher bandwidth cellular networks in LMICs could mitigate this by driving the processing to the cloud. Still, the economics of providing high-bandwidth networks to the poorest and least populated parts of the globe will still leave significant disparities in fetal monitoring care. A store-and-forward approach may offer a reasonable solution to this, with text messaging and voice calls addressing immediate issues and use of a robust remote decision-support network of trained professionals and an integrated referral mechanism (see Stroux *et al* (2016) for example)

In summary, mHealth systems bear a significant potential to provide remote perinatal care and prevent many fetal complications, by removing many barriers prevailing in LMICs. Notably, such approaches can empower the frontline healthcare workers (and perhaps even mothers) to learn, and even improve the systems, and ultimately alleviate the ‘brain drain’ in the medical field in LMICs (Cometto *et al* 2013).

## Figures and Tables

**Table 1.** Comparison of fetal cardiac monitoring methods. The first column presents a four-point ordinal scale of medical equipment cost, from low ($) to extremely high ($ $ $ $). The horizontal line indicates when, during pregnancy, the technology can be used. The color of the line indicates the time required for training operators (green: low; blue: moderate; cyan: considerable; red: high; magenta: extreme). The thickness of the line indicates the relative evidence for the utility of each technology.

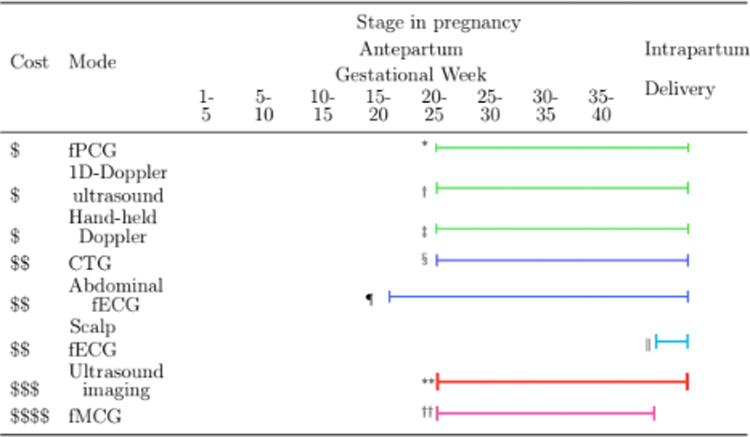

* GA *≥* 24 weeks (Várady *et al* 2003).

† GA *≥* 20 weeks (Peters *et al* 2001).

‡ GA *≥* 20 weeks (Peters *et al* 2001).

§ GA *≥* 20 weeks (Grivell *et al* 2015).

¶ GA *≥* 18 weeks (Sameni and Clifford 2010).

∥ Intrapartum (GA *≥* 36 weeks) (Norén *et al* 2006).

** GA *≥* 20 weeks (World Health Organization 2016b).

†† GA *≥* 20 weeks (Peters *et al* 2001).
